# ASP5094, a humanized monoclonal antibody against integrin alpha-9, did not show efficacy in patients with rheumatoid arthritis refractory to methotrexate: results from a phase 2a, randomized, double-blind, placebo-controlled trial

**DOI:** 10.1186/s13075-020-02336-3

**Published:** 2020-10-21

**Authors:** Tsutomu Takeuchi, Yoshiya Tanaka, Jay Erdman, Yuichiro Kaneko, Masako Saito, Chieri Higashitani, Ronald Smulders, Christopher Lademacher

**Affiliations:** 1 Keio University School of Medicine, 35 Shinanomachi, Shinjuku City, Tokyo 160-8582 Japan; 2 The First Department of Internal Medicine, University of Occupational and Environmental Health, Kitakyushu, Japan; 3 Astellas Pharma Global Development, Northbrook, IL USA; 4 Astellas Pharma, Inc., Tokyo, Japan

**Keywords:** Antibodies, Monoclonal, Humanized, Antirheumatic agents, Arthritis, Rheumatoid, Biological products, Extracellular matrix proteins, Integrins, Integrin alpha-9, human, Methotrexate, Synovial membrane, Synoviocytes

## Abstract

**Background:**

Rheumatoid arthritis (RA) is a chronic, debilitating autoimmune condition characterized by joint synovial inflammation. Current treatments include methotrexate (MTX), biologic agents, and Janus kinase (JAK) inhibitors. However, these agents are not efficacious in all patients and there are concerns regarding side effects and risk of infection as these treatments target immune-related pathways. Overexpression and activation of integrin alpha-9 (α9) on fibroblast-like synoviocytes are associated with RA disease onset and exacerbation. The humanized immunoglobulin G1 monoclonal antibody ASP5094 was designed to inhibit human α9 and is currently under investigation for the treatment of RA.

**Methods:**

This phase 2a, multicenter, randomized, placebo-controlled, double-blind, parallel-group study (NCT03257852) evaluated the efficacy, safety, and biological activity of intravenous ASP5094 10 mg/kg in patients with moderate to severe RA that was refractory to MTX. Patients received ASP5094 or placebo every 4 weeks for a total of three administrations. Both treatment groups used concomitant MTX. The primary efficacy endpoint was the proportion of patients who responded per American College of Rheumatology 50% improvement using C-reactive protein (ACR50-CRP) after 12 weeks of treatment. Biological activity of ASP5094 was assessed via pharmacokinetics and pharmacodynamics of known downstream effectors of α9. Safety was also assessed.

**Results:**

Sixty-six patients were enrolled and randomized to placebo (*n* = 33) or ASP5094 (n = 33). In the primary efficacy analysis, ACR50-CRP response rates were 6.3% and 18.2% at week 12 in the ASP5094 and placebo groups, respectively; a difference of − 11.9, which was not significant (2-sided *P value* = 0.258). No trends in ACR50 response rates were observed in subgroups based on demographics or baseline disease characteristics, and no significant differences between placebo and ASP5094 were identified in secondary efficacy or pharmacodynamic endpoints, despite achievement of target serum concentrations of ASP5094. Most treatment-emergent adverse events were mild to moderate in severity, and ASP5094 was considered safe and well tolerated overall.

**Conclusion:**

Although no notable safety signals were observed in this study, ASP5094 was not efficacious in patients with moderate to severe RA with an inadequate response to MTX.

**Trial registration:**

ClinicalTrials.gov, NCT03257852. Registered on 22 Aug. 2017

## Introduction

Rheumatoid arthritis (RA) is a chronic, systemic, inflammatory, autoimmune disease characterized by joint synovial inflammation. This condition is associated with irreversible cartilage destruction and osteolysis, resulting in pain, disability, interference in activities of daily living, and reduced quality of life [1–3]. To control synovitis and subsequent irreversible joint damage, the recommended course of treatment consists of disease-modifying antirheumatic drugs (DMARDs), initiated as soon as possible upon diagnosis of RA, with dosages adjusted to treat to a target of clinical remission, or if that is not reached, to a target of low disease activity [4].

Methotrexate (MTX) is a first-line therapy for the treatment of RA, and when the target is not achieved, biologic agents or Janus kinase (JAK) inhibitors can be added [5, 6]. Even though biologic DMARDs have several mechanisms of action, including inhibiting the activity of cytokines such as tumor necrosis factor-alpha (TNF-α) and interleukin (IL)-6 [7, 8], T-cell co-stimulatory pathways, and CD20 on B cells [9, 10], only about one half to two thirds of patients achieve clinical remission [11, 12], and these treatments are associated with the risk of infectious events [13–16]. In addition, molecular signatures characteristic of RA do not achieve normal levels following treatments targeting TNF or IL-6, even if the patient achieves clinical remission [17]. Thus, the need remains for safer and more effective treatments for RA, potentially through targeting novel mechanisms of action.

The disease process of RA involves activated fibroblast-like synoviocytes (FLS) in affected joints. These cells can lead to cartilage and bone degradation through production of matrix metalloproteinases (MMPs), upregulation of receptor activator of nuclear factor κB ligand (RANKL), production of IL-6, and recruitment and activation of proinflammatory immune cells [18–22]. Recent findings suggest distinct types of FLS may be important for chronic inflammation and bone erosion in RA and may activate lymphocytes via antigen presentation, and when present in the joint lining, be a major cause of joint destruction [23–25].

Integrin family proteins are composed of two subunits, α and β, and they bind to extracellular matrix (ECM) components and regulate a wide range of cellular responses such as migration, survival, and proliferation [26]. Integrin alpha-9 (α9) binds to ECM proteins such as tenascin-C [27], protease-cleaved osteopontin [28], and VCAM-1 [29]; contributes to cell adhesion and migration [30, 31]; and is highly expressed in synovial tissue cells, especially FLS, in patients with RA [32, 33]. This protein is also expressed on FLS of arthritic joints [34] and is overexpressed prior to onset of arthritis in mouse models [35]. Monoclonal antibodies against α9 alleviated mouse collagen-induced arthritis (CIA) [34, 36] and significantly reduced FLS-derived biomarkers [37]. Interestingly, treatment of CIA with the α9 antibody neither altered spleen cell numbers nor decreased plasma levels of anti-type II collagen antibody. Additionally, α9 antibody did not induce a mixed lymphocyte reaction or delayed type hypersensitivity reaction [37]. Thus, α9 appears to be a promising target for new therapeutics in the treatment of RA with minimal suppression of protective immunity.

ASP5094 is a humanized immunoglobulin G1 monoclonal antibody that targets human α9. Phase 1 data from healthy adult volunteers and patients with RA showed ASP5094 to be safe and well tolerated [38]. The present study investigated the efficacy, safety, pharmacokinetics (PK), and pharmacodynamics (PD) in patients with RA refractory to MTX treatment.

## Patients and methods

### Study design

**Fig. 1:**
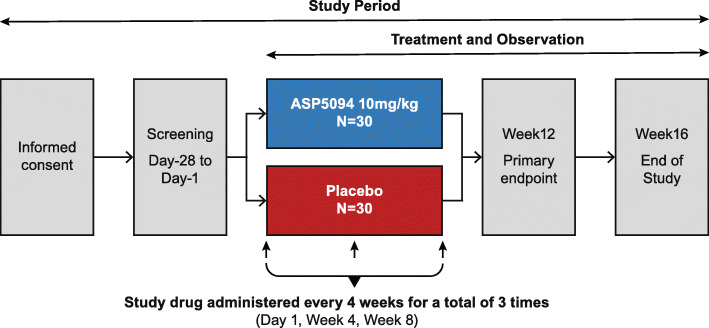
Study design

This was a phase 2a, multicenter, randomized, placebo-controlled, double-blind, parallel-group study conducted at 31 centers in Japan to evaluate the efficacy, safety, and PK of ASP5094 (10 mg/kg) in patients with moderate to severe active RA despite the use of MTX (Clinicaltrials.gov identifier: NCT03257852). Patients were randomized 1:1 to ASP5094 or placebo groups at baseline after a 28-day screening period, then received treatment with study drug every 4 weeks for a total of three administrations (day 1, week 4, and week 8; Fig. 1). Patients also received concomitant MTX (oral formulation) within the approved dose range. This study was performed in compliance with good clinical practice.

### Study patients

Eligible patients were male or female, aged 20 years or older, had a diagnosis of RA based on the 1987 American College of Rheumatology (ACR) criteria or the 2010 ACR/European League Against Rheumatism (EULAR) criteria for at least 6 months prior to screening, and met 1991 ACR Revised Criteria for the Classification of Global Functional Status in RA Class I, II, or, III. Criteria for active RA included at least six tender joints (based on 68-joint assessment), at least six swollen joints (based on 66-joint assessment), and C-reactive protein (CRP) levels exceeding 0.50 mg/dL. Eligible patients were also required to have received MTX continuously for at least 90 days prior to screening and be able to continue a stable dose of MTX from ≥ 28 days prior to screening through the study period. Patients were excluded from the study if they had ongoing infection that required antibiotics, had inflammatory arthritis other than RA (e.g., psoriatic arthritis, ankylosing spondylitis, systemic lupus erythematosus, sarcoidosis, gouty arthritis), had other articular symptoms that may have affected joint assessment, or showed evidence of autoimmune disease other than RA (excluding Sjogren’s syndrome and chronic thyroiditis), history of or concurrent malignant tumor, or other severe, progressive, or uncontrolled illness. Individuals were also excluded if they had previously shown an inadequate response to biologic DMARDs or JAK inhibitors; this exclusion criterion was implemented because the response rates of those in the refractory patient population could be lower, and targeting a more homogeneous population was a reasonable way to detect an efficacy signal in this small proof-of-concept study. Written informed consent was obtained from all patients prior to any study-related procedures.

### Study treatment

ASP5094 was prepared at the study site by unblinded staff by adding test drug to saline to be administered at a dose of 10 mg/kg in a total volume of approximately 100 mL. Placebo consisted of a matching solution of approximately 100-mL saline prepared to be indistinguishable from the active treatment. During treatment visits study patients received the prepared ASP5094 10 mg/kg solution or placebo via intravenous (IV) infusion for approximately 30 min.

### Efficacy endpoints

The primary efficacy endpoint was the response rate (i.e., proportion of patients) according to ACR 50% improvement criteria assessed using the CRP level (ACR50-CRP) at week 12. ACR50 response rates at weeks 1, 2, 4, 8, and 16, and ACR20/70 at the same time points, plus week 12 assessments were determined as secondary endpoints. Additional secondary endpoints included change from baseline in the Disease Activity Score (DAS) 28-CRP or DAS28-erythrocyte sedimentation rate (ESR) score, Simplified Disease Activity Index (SDAI) score, and Clinical Disease Activity Index (CDAI) score through week 16, as well as rates of remission and low disease activity based on DAS28 score (< 2.6 and ≤ 3.2, respectively), “good” or “moderate” response according to EULAR criteria, and remission based on ACR/EULAR score.

### Pharmacokinetics

Blood samples were collected at baseline and at each scheduled visit to determine serum concentrations of ASP5094. Samples were collected once each during the visits on week 1, week 2, week 12/discontinuation, and week 16/discontinuation, then 4 weeks later; and twice (before and after study drug administration) on day 1 (baseline), week 4, and week 8. The blood samples taken after study drug administration were generally collected within 15 min after the end of treatment from the patient’s arm opposite from where study drug was administered. Serum samples were diluted to 1:100 in casein-containing sample buffer and incubated on a microtiter plate, MULTI-ARRAY 96-Well Plate (Standard, Meso Scale Diagnostics, LLC), coated with an antibody to capture ASP5094, then were washed three times with PBS containing 0.05% Tween 20 and incubated with a luminescent detection antibody that was quantified and analyzed by electrochemiluminescence immunoassay (ELCIA), using SECTOR (Meso Scale Discovery, Rockville, MD). ASP5094-specific capture, as well as biotin-labeled secondary antibodies, were purposefully created for this assay and are not commercially available.

### Pharmacodynamics

Pharmacodynamic assays were performed to assess the activity of ASP5094 on the known downstream effectors of α9 in RA of TNF-α, MMP-3, and IL-6, which were measured at baseline and each scheduled visit. These serum concentrations were measured by commercially available kits as follows: TNF-α; ELISA, Quantikine HS ELISA (R&D Systems), MMP-3; LTIA, Panaclear MMP-3: Latex (Sekisui Medical), IL-6; CLEIA, IL-6 LPG Immunoreaction Cartridges (Fujirebio). Levels of putative α9 ligands tenascin (TNC)-C (large variant of tenascin-C, termed FNIII-B variant), vascular cell adhesion molecule (VCAM)-1, and osteopontin (OPN; full-length and thrombin-cleaved) were determined at baseline and week 12/discontinuation and week 16/discontinuation, then 4 weeks later for exploratory evaluation of ASP5094 on potential biomarkers. Plasma concentrations of TNC-C, VCAM-1, and OPN were measured by a commercially available ELISA kit as follows: TNC-C; Tn-C large [FNIII-B] assay kit (Immuno-Biological Laboratories), VCAM-1; Human sVCAM1/CD106 Quantikine ELISA kit; (R&D Systems), OPN full-length; Human osteopontin Quantikine ELISA kit (R&D Systems), cleaved-OPN; Human osteopontin N-half assay kit (Immuno-Biological Laboratories).

### Safety

Safety was assessed via incidence of treatment-emergent adverse events (TEAEs) over the duration of the study, and by electrocardiogram, vital signs, bodyweight and laboratory assessments comprising hematology, biochemistry, and urinalysis at each scheduled visit.

### Sample size determination and statistical analyses

Based on findings of previous clinical studies in RA [11, 39–41], and assuming ACR50 response rates at week 12 would be 10% in the placebo group and 50% in the ASP5094 group, a sample size of 26 patients per group was expected to provide 90% power to detect a difference between the groups at a 2-sided significance level of 0.10. Therefore, 60 patients were planned to be randomized in a 1:1 ratio to ASP5094 (*n* = 30) or placebo (*n* = 30).

### Statistical analyses

Efficacy analyses were based on the full analysis set (FAS), defined as all patients who received at least one dose of study drug and had at least one efficacy measurement after study drug administration. The safety analysis set (SAF) used for safety analyses consists of all patients who took at least one dose of study drug. The PK and PD analyses were based on corresponding analysis sets that comprised patients who received study drug and from whom at least one PK and PD analysis sample, respectively, was collected after administration.

For comparisons of binary variables, Fisher’s exact test was used with a significance level of 10% (2-sided), unless otherwise indicated. Differences of response rates between treatment groups and 2-sided 90% confidence interval (based on the normal approximation) were also calculated. Continuous variables were compared using an analysis of covariance, with treatment group as a factor and baseline score as a covariate; mean and standard deviation (SD) of actual values and changes from baseline were displayed. For missing data, nonresponder imputation (NRI) was used for the primary analysis and last observation carried forward (LOCF) for all secondary endpoints.

## Results

### Patients

**Fig. 2:**
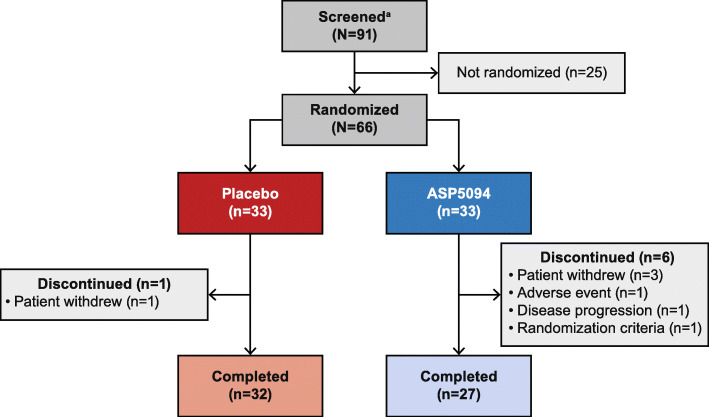
Patient disposition. ^a^Provided informed consent

Among 91 recruited patients, 66 were enrolled and randomized to placebo (*n* = 33) or ASP5094 (*n* = 33) and comprised the SAF. A total of 59 patients (89.4%) completed the study; 1 (3.0%) and six (18.2%) patients from the placebo and ASP5094 groups, respectively, discontinued prior to study end (Fig. 2). The FAS included 65 patients (placebo, *n* = 33; ASP5094, *n* = 32) as one patient in the ASP5094 group was excluded from the analyses for having no data for the efficacy endpoints.

The mean age of randomized patients was 56.3 years and 47 (70.8%) were female. Demographic characteristics and baseline disease activity were similar across treatment groups (Table 1).

**Table 1:** Patient demographics, disease history, and baseline disease characteristics

Parameter		
**Demographics and disease history**^**a**^	**Placebo (*n*** **= 33)**	**ASP5094 (*n*** **= 33)**
Age, years, mean ± SD	57.9 ± 9.1	55.7 ± 13.8
Median (range)	57.0 (41–77)	55.5 (30–77)
≥ 65 years, *n* (%)	9 (27.3%)	10 (30.3%)
Female, *n* (%)	26 (78.8%)	21 (63.6%)
Weight, kg, mean ± SD	57.82 ± 11.13	62.83 ± 15.16
BMI, kg/m^2^, mean ± SD	22.81 ± 4.09	24.33 ± 4.20
Duration of RA, years, mean ± SD	9.28 ± 8.27	9.87 ± 9.41
MTX dose at screening, mg/week, mean ± SD	10.08 ± 2.86	9.65 ± 3.46
> 0 ≤ 8 mg/week, *n* (%)	10 (30.3)	15 (45.5)
> 8 ≤ 12 mg/week, *n* (%)	18 (54.5)	11 (33.3)
> 12 mg/week, *n* (%)	5 (15.2)	7 (21.2)
**Baseline disease activity, mean ± SD**^**b**^	**Placebo (*n*** **= 33)**	**ASP5094 (*n*** **= 32)**
Tender joint count (68 joints)	12.3 ± 5.7	12.6 ± 5.1
Swollen joint count (66 joints)	11.2 ± 5.1	11.5 ± 4.6
Patient’s global assessment of arthritis pain^a^	42.58 ± 23.20	54.42 ± 23.13
Patient’s global assessment of arthritis^a^	47.94 ± 22.36	51.42 ± 23.83
Physician’s global assessment of arthritis^a^	51.82 ± 16.20	52.89 ± 15.78
CRP, mg/dL	1.406 ± 1.031	1.668 ± 1.418
ESR, mm/h	42.48 ± 21.26	43.09 ± 24.55
DAS28-CRP score	4.98 ± 0.68	5.15 ± 0.61
DAS28-ESR score	5.66 ± 0.79	5.67 ± 0.82
SDAI score	28.90 ± 8.39	30.32 ± 8.21
CDAI score	27.49 ± 8.17	28.65 ± 8.10
HAQ-DI score	0.750 ± 0.537	0.754 ± 0.712

^a^Safety analysis set

^b^Full analysis set

^c^Based on 100-mm analog scale

*BMI* body mass index, *CDAI* Clinical Disease Activity Index, *CRP* C-reactive protein, *DAS28* Disease Activity Score in 28 Joints, *ESR* erythrocyte sedimentation rate, *FAS* full analysis set, *HAQ-DI* Health Assessment Questionnaire-Disability Index, *MTX* methotrexate, *RA* rheumatoid arthritis, *SD* standard deviation, *SDAI*, Simplified Disease Activity Index

### Efficacy

For the primary efficacy endpoint of ACR50-CRP response rates at week 12, the proportion of patients in ASP5094 group (2/32; 6.3%) was not higher than in the placebo group (6/33; 18.2%). This represented a difference of − 11.9% between groups, which was not statistically significant (*P* = 0.258; Table 2). Rates of ACR50-CRP response over time are provided in Supplemental Fig. 1. Subgroup analyses were performed to determine whether particular demographic or disease characteristics, such as age, sex, MTX dose, duration of RA, and baseline disease activity, may affect treatment outcomes. Subgroup analysis did not reveal any obvious trends in ACR50 response rate between ASP5094 and placebo (Supplemental Table 1).

**Table 2:** ACR response rates at week 12

	Placebo (***n*** = 33)	ASP5094 (***n*** = 32)
ACR50 responders, *n* (%)^a^	6 (18.2)	2 (6.3)
Difference (90% CI)	−11.9 (−25.0, 1.2)	
*P* value^b^	0.258	
ACR20 responders, *n* (%)^c^	16 (48.5)	12 (37.5)
Difference (90% CI)	−11.0 (− 31.1, 9.1)	
*P* value^b^	0.455	
ACR70 responders, *n* (%)^c^	2 (6.1)	1 (3.1)
Difference (90% CI)	−2.9 (−11.4, 5.6)	
*P* value^b^	1.000	

^a^Primary efficacy endpoint, assessed via nonresponder imputation

^b^*P* values based on Fisher’s exact test

^c^Secondary efficacy endpoint, assessed via last observation carried forward technique

*ACR* American College of Rheumatology, *CI* confidence interval

Secondary ACR efficacy endpoints at week 12 included ACR20 and ACR70 response rates. Similar to the results observed with ACR50, the proportion of patients in the ASP5094 group who achieved these endpoints was not higher than in the placebo group (Table 2).

**Fig. 3:**
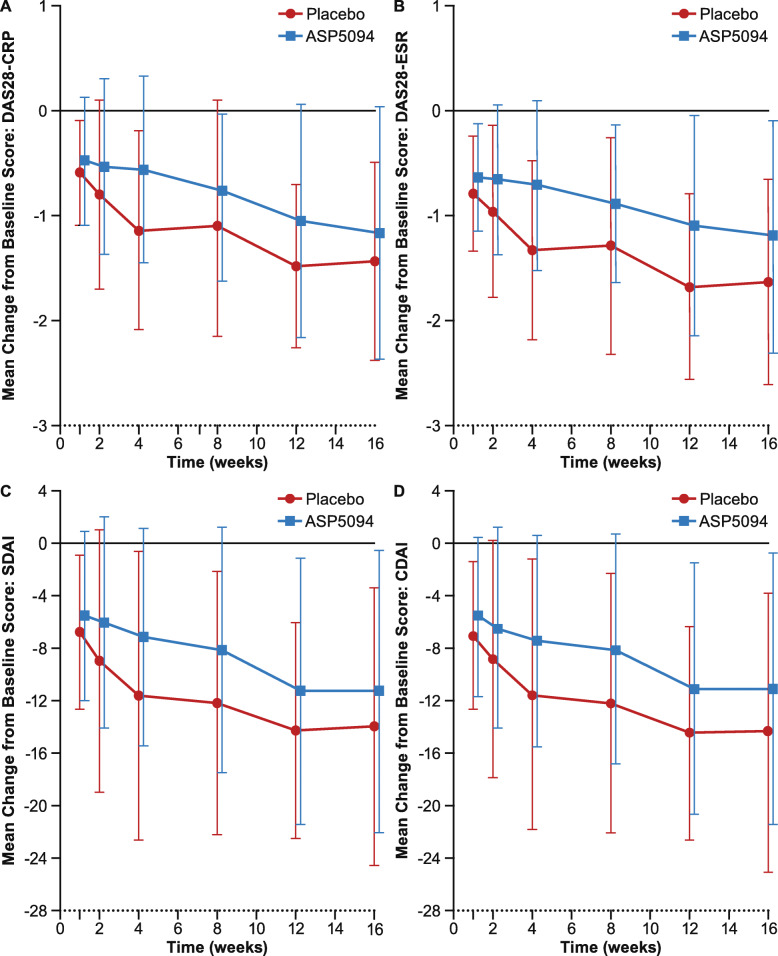
Change from baseline in **a** DAS28-CRP, **b** DAS28-ESR, **c** SDAI, and **d** CDAI scores over time. CDAI, Clinical Disease Activity Index; CRP, c-reactive protein; DAS28, Disease Activity Score in 28 joints; ESR, erythrocyte sedimentation rate; SDAI, Simplified Disease Activity Index

When additional secondary endpoints were examined, findings failed to demonstrate significant improvement in ASP5094 across all timepoints. Specifically, the change from baseline in DAS28-CRP (Fig. 3a), DAS28-ESR (Fig. 3b), SDAI (Fig. 3c), and CDAI (Fig. 3d) values demonstrate that ASP5094 did not lead to statistically significant or clinically meaningful improvements, compared with placebo.

### Pharmacokinetics/pharmacodynamics

A summary of serum concentrations of ASP5094 following 10 mg/kg dose administration on day 1 and at week 4 and week 8 is presented in Table 3. The peak mean serum ASP5094 concentration was achieved at the end of infusion. A trend of increasing mean serum ASP5094 concentrations prior to dosing was observed at each successive treatment visit, suggesting a steady state of ASP5094 serum concentration was not achieved after three doses.

**Table 3:** ASP5094 serum concentrations by time and dosing

	Baseline (day 1)		Week 1 (***n*** = 32)	Week 2 (***n*** = 32)	Week 4		Week 8		Week 12 (***n*** = 27)	Week 16 (***n*** = 27)
	Pre (***n*** = 33)	Post (***n*** = 33)			Pre (***n*** = 31)	Post (***n*** = 27)	Pre (***n*** = 27)	Post (***n*** = 27)		
Mean ± SD	0 ± NA	242 ± 42.9	101 ± 17.2	64.2 ± 10.5	27.2 ± 10.2	270 ± 42.4	38.4 ± 13.8	292 ± 39.5	43.1 ± 16.7	5.55 ± 6.5
Median (range)	0 (0–0)	240 (139–345)	102 (67–149)	63.6 (43.3–85)	26.8 (10.6–51.6)	264 (198–364)	35.3 (13.3–67)	289 (227–372)	42.1 (11.6–91.6)	3.95 (0–26.6)

*NA* not available, *SD* standard deviation

The mean change and percent changes from baseline in TNF-α, MMP-3, IL-6, TNC-C, VCAM-1, and OPN (full-length and thrombin-cleaved) at week 12 are summarized in Supplemental Table 2. The change from baseline in PD endpoints generally showed large variability, and there were no obvious differences in any of these endpoints between the ASP5094 and placebo groups.

### Safety

The incidence of TEAEs was 39.4% and 60.6% in the placebo and ASP5094 groups, respectively (Table 4). The only TEAEs with an incidence ≥ 10% in any treatment group were worsening of RA (placebo: *n* = 4 [12.1%]; ASP5094: *n* = 6 [18.2%]) and viral upper respiratory tract infection (placebo: *n* = 3 [9.1%]; ASP5094: *n* = 4 [12.1%]). Most TEAEs were mild to moderate in severity, except for severe events of bronchitis and influenza in one patient in the ASP5094 group. The incidence of drug-related TEAEs was 6.1% and 15.2% in the placebo and ASP5094 groups, respectively. One patient in the placebo group experienced a serious TEAE (spinal compression fracture) while two patients in the ASP5094 group experienced serious TEAEs (severe bronchitis and influenza considered to be possibly drug related in one patient, and two events of tendon rupture in the other patient who used a steroid). The events of tendon rupture were considered attributable to joint deformation caused by joint destruction and daily motion and were assessed to be not related to study drug by the physician. No deaths were reported during the study. No clinically significant laboratory abnormalities were detected in either group, and there were no remarkable differences in changes in laboratory values between groups.

**Table 4:** Summary of TEAEs

*n* (%)	Placebo (***n*** = 33)	ASP5094 (***n*** = 33)
**TEAE**	13 (39.4%)	20 (60.6%)
Mild	9 (27.3%)	13 (39.4%)
Moderate	4 (12.1%)	6 (18.2%)
Severe	0	1 (3.0%)
Drug-related TEAE	2 (6.1%)	5 (15.2%)
Serious TEAE	1 (3.0%)	2 (6.1%)
Drug-related serious TEAE	0	1 (3.0%)
TEAE leading to permanent discontinuation	0	2 (6.1%)
Drug-related TEAE leading to discontinuation	0	1 (3.0%)
Serious TEAE leading to permanent discontinuation	0	0
Death	0	0
**TEAEs experienced by > 1 patient overall**		
Rheumatoid arthritis	4 (12.1%)	6 (18.2%)
Viral upper respiratory tract infection	3 (9.1%)	4 (12.1%)
Constipation	1 (3.0%)	3 (9.1%)
Influenza	1 (3.0%)	2 (6.1%)
Chest discomfort	2 (6.1%)	1 (3.0%)
Cystitis	1 (3.0%)	1 (3.0%)
Pneumonia	1 (3.0%)	1 (3.0%)

*TEAE* treatment-emergent adverse event

## Discussion

The development of biologic agents targeting cytokines, T cells, and B cells marked a revolutionary change in the treatment of RA. However, not all patients achieve treatment targets, and some experience adverse events such as severe infections, highlighting the need for new therapeutic targets. Accumulating evidence supports activation and/or increased expression of α9 as a key mechanism in the RA disease process [20, 32, 42] including α9 overexpression on FLS in rheumatic joints preceding the onset of arthritis [18, 19, 35], and the activation of α9 stimulating transformation of FLS into a pathologic state that includes hyperplastic and proinflammatory activity [33]. Moreover, monoclonal antibodies against α9 suppressed arthritis development and reduced FLS-derived biomarkers while sparing systemic immune activity in mice [37]. Thus, α9 was identified as a potential target for therapeutic development in RA.

In the current study, we investigated the clinical efficacy, safety, and PK and PD effects of the monoclonal α9 blocking antibody ASP5094 in patients with moderate to severe active RA. Our results revealed that 10 mg/kg ASP5094 was not more efficacious than placebo plus MTX at improving signs or symptoms of RA. The primary efficacy endpoint of ACR50 response rate at week 12 showed no clinically meaningful or statistically significant improvement with ASP5094 versus placebo, and no meaningful differences between treatment groups were observed in any secondary endpoints. Exploratory biomarkers were highly variable and there was no clear indication of PD modulation by ASP5094. We also did not observe any clear differences or trends between treatments within subgroups classified by demographics or potential stratification biomarkers, further indicating a lack of clinical efficacy by ASP5094.

The dose of 10 mg/kg tested in this study was chosen based on the findings in a previous phase 1 study [38, 43] in which binding of ASP5094 to α9 on neutrophils was assessed via percent receptor occupancy (%RO). In that study, 3 mg/kg ASP5094 led to RO above 80% for 85 days, and above 90% for 141 days with 10 mg/kg ASP5094. RO was not assessed in the current study, yet minimum serum ASP5094 concentrations ranged from 10.6 to 13.3 μg/mL at the end of each dosing period and was 11.6 μg/mL at week 12, which was more than 100-fold higher than the mean day 85 predose level (3 mg/kg dose group) in the previous study. Therefore, the target serum ASP5094 level was likely achieved in the current study, and the lack of clinical effectiveness or PD effects occurred while serum ASP5094 concentrations were within the expected range.

There are several potential explanations for the lack of efficacy we observed. First, the favorable preclinical data for ASP5094 that supported clinical investigation was obtained using a mouse model of acute arthritis in which administration of anti-mouse α9 antibody suppressed type II collagen-induced arthritis in both prophylactic and therapeutic regimens [37]. In contrast with the mouse, increased interstitial pressure by hyperplasia and extracellular matrix deposition in humans with RA might prevent drug diffusion from blood vessels to synovial tissues. Although our PK results suggest adequate serum levels of ASP5094 were achieved, these measurements were performed in peripheral blood, which does not necessarily confirm that ASP5094 occupied and suppressed α9 in the target tissue. A discrepancy between circulating ASP5094 and target tissue ASP5094 could explain the lack of PD effects; however, these data are not feasible to obtain in patients. Second, extremely high local concentrations of multiple α9 ligands such as OPN, tenascin-C, or VCAM-1 may compete with ASP5094 for binding to α9 integrin. In our previous study, ASP5094 neutralized binding of α9 to its ligand with an IC50 value of around 1 ng/mL, indicating that the antibody had good neutralizing potency in vitro ([33], Fig. 7A). However, in another study, a higher concentration of ASP5094 was required to suppress in vitro action of FLS derived from RA patients ([33], Fig. 7C). One possible explanation for this discrepancy is that huge amounts of ligands in the system produced by FLS affected the neutralizing activity of ASP5094. Additionally, there are data that suggest OPN levels in synovial fluid are markedly elevated compared with levels in the blood in patients with RA [43], and these may out-compete α9 for ASP5094 binding. Moreover, increased levels of locally expressed α9 might absorb a significant portion of circulating ASP5094, thereby depleting the amount available for binding in the synovium. Finally, the disease model in the aforementioned preclinical studies was of acute arthritis, which may not adequately represent the complex nature of chronic human RA. It is possible that the α9 neutralization that suppressed synovium activation and yielded positive results in the mouse model is not sufficient by itself to treat human RA where direct modulation of immune-pathway activation through additional targets may also be required. Thus, α9 alone may not be an effective target in the treatment of RA.

Although the incidence of overall and drug-related TEAEs was higher in the ASP5094 group, the overall incidence of severe and/or serious TEAEs was low. Overall, no notable safety signals were observed in this study, but further safety data will be needed in order to ascertain the overall safety profile of ASP5094 in patients with RA.

Single-cell analysis data from synovial tissue samples were published in 2019 by Zhang et al. [25]. Based on the information provided by these types of innovative technologies, it might be interesting to consider testing ASP5094 in patients specifically characterized by α9 overexpression.

## Conclusions

In this study of patients with moderate to severe active RA refractory to MTX treatment, 10 mg/kg ASP5094 was not effective for the treatment of RA. Despite the lack of efficacy, no notable safety signals were observed in this study. Results of PK and PD assessments suggested that the expected serum levels of ASP5094 were achieved, yet expected molecular target effects were not observed. These findings may suggest target site exposure to ASP5094 in RA tissue was insufficient, or that targeting integrin α9 alone may not be adequate to deactivate FLS and thus improve RA.

## Supplementary information

**Additional file 1: Supplemental Table 1.** Analyses of ACR50-CRP response at Week 12 by baseline characteristic (NRI; FAS). **Supplemental Table 2.** Result, change, and percent change from baseline in pharmacodynamics at Week 12. **Supplemental Fig.** 1 ACR50-CRP response over time.

## Data Availability

Researchers may request access to anonymized participant level data, trial level data, and protocols from Astellas sponsored clinical trials at www.clinicalstudydatarequest.com. For the Astellas criteria on data sharing see https://clinicalstudydatarequest.com/Study-Sponsors/Study-Sponsors-Astellas.aspx.

## Abbreviations

α9: Integrin alpha-9
ACR: American College of Rheumatology
ACR50-CRP: American College of Rheumatology 50% improvement using C-reactive protein
BMI: Body mass index
CDAI: Clinical Disease Activity Index
CRP: C-reactive protein
DMARDs: Disease-modifying antirheumatic drugs
ELISA: Enzyme-linked immunosorbent assay
ESR: Erythrocyte sedimentation rate
EULAR: European League Against Rheumatism
FAS: Full analysis set
FLS: Fibroblast-like synoviocytes
IV: Intravenous
JAK: Janus kinase
LOCF: Last observation carried forward
MMP: Matrix metalloproteinases
MTX: Methotrexate
NRI: Nonresponder imputation
OPN: Osteopontin
PD: Pharmacodynamic
PK: Pharmacokinetic
RA: Rheumatoid arthritis
RO: Receptor occupancy
SD: Standard deviation
SDAI: Simplified Disease Activity Index
TNC-C: Tenascin-C
TEAE: Treatment-emergent adverse event
VCAM: Vascular cell adhesion molecule
