# Supplementary material for: ASP5094, a humanized monoclonal antibody against integrin alpha-9, did not show efficacy in patients with rheumatoid arthritis refractory to methotrexate: results from a phase 2a, randomized, double-blind, placebo-controlled trial
Source: Arthritis Res Ther. 2020 Oct 21;22:252. doi: 10.1186/s13075-020-02336-3 (PMC7579887; doi:10.1186/s13075-020-02336-3)
Supplement: Supplementary file 1 — Additional file 1: Supplemental Table 1. Analyses of ACR50-CRP response at Week 12 by baseline characteristic (NRI; FAS). Supplemental Table 2. Result, change, and percent change from baseline in pharmacodynamics at Week 12. Supplemental Fig. 1 ACR50-CRP response over time. [file 13075_2020_2336_MOESM1_ESM.docx]

# SUPPLEMENTARY MATERIAL

| **Supplemental Table 1. Analyses of ACR50-CRP response at Week 12 by baseline characteristic (NRI; FAS)** | | | | | |
| --- | --- | --- | --- | --- | --- |
| **Subgroup** | **Category** | **Treatment group** | **n** | **Responder, n (%)** | **Difference, % (90% CI)** |
| **Age** | <65 years | Placebo | 24 | 3 (12.5%) | -3.8 (-18.5, 10.9) |
|  |  | ASP5094 | 23 | 2 (8.7%) |  |
|  | ≥65 years | Placebo | 9 | 3 (33.3%) | -33.3 (-59.2, -7.5) |
|  |  | ASP5094 | 9 | 0 |  |
| **MTX dose (screening)** | ≤8 mg/week | Placebo | 10 | 1 (10.0%) | -2.9 (-22.1, 16.4) |
|  |  | ASP5094 | 14 | 1 (7.1%) |  |
|  | >8 mg/week | Placebo | 23 | 5 (21.7%) | -16.2 (-32.9, 0.5) |
|  |  | ASP5094 | 18 | 1 (5.6%) |  |
| **Prior biologic DMARD or JAK inhibitors** | Yes | Placebo | 5 | 0 | 14.3 (-7.5, 36.0) |
|  |  | ASP5094 | 7 | 1 (14.3%) |  |
|  | No | Placebo | 28 | 6 (21.4%) | -17.4 (-31.7, -3.1) |
|  |  | ASP5094 | 25 | 1 (4.0%) |  |
| **RA duration** | <10 years | Placebo | 22 | 4 (18.2%) | -18.2 (-31.7, -4.7) |
|  |  | ASP5094 | 22 | 0 |  |
|  | ≥10 years | Placebo | 11 | 2 (18.2%) | 1.8 (-26.4, 30.1) |
|  |  | ASP5094 | 10 | 2 (20.0%) |  |
| **DAS28-CRP** | ≤5.1 | Placebo | 19 | 3 (15.8%) | -1.5 (-22.1, 19.1) |
|  |  | ASP5094 | 14 | 2 (14.3%) |  |
|  | >5.1 | Placebo | 14 | 3 (21.4%) | -21.4 (-39.5, -3.4) |
|  |  | ASP5094 | 18 | 0 |  |
| **DAS28-ESR** | ≤5.1 | Placebo | 9 | 2 (22.2%) | -5.6 (-39.4, 28.3) |
|  |  | ASP5094 | 6 | 1 (16.7%) |  |
|  | >5.1 | Placebo | 24 | 4 (16.7%) | -12.8 (-26.8, 1.1) |
|  |  | ASP5094 | 26 | 1 (3.8%) |  |
| **CRP (ng/dL)** | <1.0 | Placebo | 14 | 2 (14.3%) | -1.8 (-26.4, 22.8) |
|  |  | ASP5094 | 8 | 1 (12.5%) |  |
|  | ≥1.0 | Placebo | 19 | 4 (21.1%) | -16.9 (-33.7, -0.1) |
|  |  | ASP5094 | 24 | 1 (4.2%) |  |
| **TNF-α** | ≤Median | Placebo | 28 | 4 (14.3%) | -9.3 (-22.8, 4.2) |
|  |  | ASP5094 | 20 | 1 (5.0%) |  |
|  | >Median | Placebo | 5 | 2 (40.0%) | -31.7 (-70.0, 6.7) |
|  |  | ASP5094 | 12 | 1 (8.3%) |  |
| **MMP-3** | ≤Median | Placebo | 18 | 3 (16.7%) | -16.7 (-31.1, -2.2) |
|  |  | ASP5094 | 15 | 0 |  |
|  | >Median | Placebo | 15 | 3 (20.0%) | -8.2 (-29.5, 13.1) |
|  |  | ASP5094 | 17 | 2 (11.8%) |  |
| **IL-6** | ≤Median | Placebo | 18 | 3 (16.7%) | -10.0 (-27.9, 7.9) |
|  |  | ASP5094 | 15 | 1 (6.7%) |  |
|  | >Median | Placebo | 15 | 3 (20.0%) | -14.1 (-33.5, 5.3) |
|  |  | ASP5094 | 17 | 1 (5.9%) |  |
| **TNC-C** | ≤Median | Placebo | 11 | 1 (9.1%) | -4.5 (-20.6, 11.5) |
|  |  | ASP5094 | 22 | 1 (4.5%) |  |
|  | >Median | Placebo | 22 | 5 (22.7%) | -12.7 (-34.2, 8.7) |
|  |  | ASP5094 | 10 | 1 (10.0%) |  |
| **VCAM-1** | ≤Median | Placebo | 14 | 2 (14.3%) | -14.3 (-29.7, 1.1) |
|  |  | ASP5094 | 19 | 0 |  |
|  | >Median | Placebo | 19 | 4 (21.1%) | -5.7 (-28.2, 16.9) |
|  |  | ASP5094 | 13 | 2 (15.4%) |  |
| **OPN (full-length)** | ≤Median | Placebo | 22 | 4 (18.2%) | -18.2 (-31.7, -4.7) |
|  |  | ASP5094 | 11 | 0 |  |
|  | >Median | Placebo | 11 | 2 (18.2%) | -8.7 (-30.5, 13.2) |
|  |  | ASP5094 | 21 | 2 (9.5%) |  |
| **OPN (thrombin-cleaved)** | ≤Median | Placebo | 17 | 3 (17.6%) | -11.4 (-29.6, 6.8) |
|  |  | ASP5094 | 16 | 1 (6.3%) |  |
|  | >Median | Placebo | 16 | 3 (18.8%) | -12.5 (-31.4, 6.4) |
|  |  | ASP5094 | 16 | 1 (6.3%) |  |
| Abbreviations: ACR, American College of Rheumatology; ACR50-CRP, American College of Rheumatology 50% improvement assessed using the C-reactive protein level; CI, confidence interval; CRP, C-reactive protein; DAS28, Disease Activity Score-28 Joints; DMARD, disease-modifying antirheumatic drug; ESR, erythrocyte sedimentation rate; FAS, full analysis set; IL, interleukin; JAK, Janus kinase; MMP, matrix metalloproteinase; MTX, methotrexate; NRI, nonresponder imputation; OPN, osteopontin; RA, rheumatoid arthritis; TNC, tenascin; TNF, tumor necrosis factor; VCAM, vascular cell adhesion molecule. | | | | | |

| **Supplemental Table 2. Result, change, and percent change from baseline in pharmacodynamics at Week 12** | | | | | | |
| --- | --- | --- | --- | --- | --- | --- |
| **Parameter** | **Group^a^** | **Visit** | **Result** | | | |
|  |  |  | **Mean ± SD** | **Q1** | **Median** | **Q3** |
| **TNFα (IU/L)** | **Placebo** | Baseline (n=33) | 0.039 ± 0.016 | 0.030 | 0.040 | 0.040 |
|  |  | Week 12 (n=33) | 0.045 ± 0.033 | 0.030 | 0.040 | 0.050 |
|  |  | Change from baseline at Week 12 (n=33) | 0.006 ± 0.033 | -0.010 | 0.000 | 0.010 |
|  |  | Percent change from baseline at Week 12 (n=33) | 22.102 ± 82.812 | -16.667 | 0.000 | 33.333 |
|  | **ASP5094** | Baseline (n=32) | 0.111 ± 0.396 | 0.030 | 0.040 | 0.050 |
|  |  | Week 12 (n=27) | 0.102 ± 0.312 | 0.030 | 0.040 | 0.050 |
|  |  | Change from baseline at Week 12 (n=27) | -0.023 ± 0.120 | 0.000 | 0.000 | 0.000 |
|  |  | Percent change from baseline at Week 12 (n=27) | 0.598 ± 19.852 | 0.000 | 0.000 | 0.000 |
| **MMP (ng/mL)** | **Placebo** | Baseline (n=33) | 194.11 ± 177.39 | 82.30 | 144.00 | 195.00 |
|  |  | Week 12 (n=33) | 196.49 ± 170.68 | 88.30 | 126.00 | 219.00 |
|  |  | Change from baseline at Week 12 (n=33) | 2.38 ± 177.97 | -34.00 | -12.60 | 27.00 |
|  |  | Percent change from baseline at Week 12 (n=33) | 15.91 ± 75.57 | -28.74 | -13.44 | 27.56 |
|  | **ASP5094** | Baseline (n=32) | 213.31 ± 141.60 | 115.50 | 169.50 | 293.50 |
|  |  | Week 12 (n=27) | 161.92 ± 118.12 | 72.70 | 107.00 | 231.00 |
|  |  | Change from baseline at Week 12 (n=27) | -36.64 ± 85.77 | -60.00 | -14.00 | 10.10 |
|  |  | Percent change from baseline at Week 12 (n=27) | -13.64 ± 29.51 | -34.90 | -12.12 | 11.40 |
| **IL-6 (pg/mL)** | **Placebo** | Baseline (n=33) | 27.50 ± 44.69 | 4.80 | 13.20 | 33.20 |
|  |  | Week 12 (n=33) | 27.96 ± 60.09 | 5.30 | 10.90 | 24.90 |
|  |  | Change from baseline at Week 12 (n=33) | 0.46 ± 73.28 | -14.10 | -1.60 | 6.90 |
|  |  | Percent change from baseline at Week 12 (n=33) | 270.89 ± 1342.64 | -49.85 | -24.00 | 78.57 |
|  | **ASP5094** | Baseline (n=32) | 22.15 ± 18.67 | 8.60 | 15.80 | 31.70 |
|  |  | Week 12 (n=27) | 15.57 ± 18.64 | 5.50 | 9.60 | 15.30 |
|  |  | Change from baseline at Week 12 (n=27) | -4.58 ± 20.58 | -9.90 | -1.50 | 3.40 |
|  |  | Percent change from baseline at Week 12 (n=27) | -4.34 ± 58.65 | -61.58 | -12.07 | 51.64 |
| **TNC-C (ng/mL)** | **Placebo** | Baseline (n=33) | 826.7 ± 306.0 | 615.0 | 743.0 | 967.0 |
|  |  | Week 12 (n=33) | 803.0 ± 430.1 | 558.0 | 659.0 | 917.0 |
|  |  | Change from baseline at Week 12 (n=33) | -23.7 ± 362.8 | -218.0 | -95.0 | 128.0 |
|  |  | Percent change from baseline at Week 12 (n=33) | 1.3 ± 39.2 | -24.90 | -11.30 | 20.80 |
|  | **ASP5094** | Baseline (n=32) | 661.1 ± 201.4 | 507.5 | 617.0 | 780.0 |
|  |  | Week 12 (n=27) | 576.4 ± 209.7 | 421.0 | 528.0 | 660.0 |
|  |  | Change from baseline at Week 12 (n=27) | -51.9 ± 160.3 | -179.0 | -58.0 | 61.0 |
|  |  | Percent change from baseline at Week 12 (n=27) | -7.2 ± 25.2 | -24.9 | -15.7 | 8.7 |
| **VCAM-1 (ng/mL)** | **Placebo** | Baseline (n=33) | 699.8 ± 181.9 | 565.0 | 715.0 | 787.0 |
|  |  | Week 12 (n=33) | 727.7 ± 181.9 | 594.0 | 722.0 | 828.0 |
|  |  | Change from baseline at Week 12 (n=33) | 27.9 ± 71.2 | 1.0 | 39.0 | 72.0 |
|  |  | Percent change from baseline at Week 12 (n=33) | 4.7 ± 9.8 | 0.2 | 4.8 | 10.6 |
|  | **ASP5094** | Baseline (n=32) | 689.1 ± 212.1 | 575.5 | 629.5 | 770.0 |
|  |  | Week 12 (n=27) | 727.3 ± 242.9 | 575.0 | 665.0 | 790.0 |
|  |  | Change from baseline at Week 12 (n=27) | 33.3 ± 86.9 | -18.0 | 23.0 | 64.0 |
|  |  | Percent change from baseline at Week 12 (n=27) | 5.4 ± 14.4 | -3.0 | 3.3 | 10.2 |
| **OPN, full-length (ng/mL)** | **Placebo** | Baseline (n=33) | 80.13 ± 28.87 | 58.40 | 75.10 | 102.00 |
|  |  | Week 12 (n=33) | 82.43 ± 33.71 | 62.70 | 82.60 | 91.90 |
|  |  | Change from baseline at Week 12 (n=33) | 2.29 ± 26.18 | -9.60 | 0.70 | 11.00 |
|  |  | Percent change from baseline at Week 12 (n=33) | 10.40 ± 63.56 | -12.47 | 1.30 | 10.79 |
|  | **ASP5094** | Baseline (n=32) | 91.96 ± 28.42 | 72.25 | 91.65 | 106.00 |
|  |  | Week 12 (n=27) | 87.14 ± 30.43 | 63.60 | 84.00 | 103.00 |
|  |  | Change from baseline at Week 12 (n=27) | -3.93 ± 16.70 | -14.10 | -7.40 | 1.30 |
|  |  | Percent change from baseline at Week 12 (n=27) | -3.39 ± 18.37 | -14.59 | -8.10 | 1.67 |
| **OPN, thrombin-cleaved (pmol/L)** | **Placebo** | Baseline (n=33) | 45.86 ± 119.10 | 0.00 | 0.00 | 29.90 |
|  |  | Week 12 (n=33) | 91.40 ± 268.22 | 0.00 | 0.00 | 17.60 |
|  |  | Change from baseline at Week 12 (n=33) | 45.54 ± 293.18 | -18.30 | 0.00 | 3.00 |
|  |  | Percent change from baseline at Week 12 (n=16) | -40.35 ± 123.48 | -100.00 | -100.00 | -4.62 |
|  | **ASP5094** | Baseline (n=32) | 27.24 ± 41.23 | 0.00 | 14.00 | 31.75 |
|  |  | Week 12 (n=27) | 24.32 ± 48.86 | 0.00 | 0.00 | 19.00 |
|  |  | Change from baseline at Week 12 (n=27) | -5.04 ± 57.19 | -21.20 | 0.00 | 0.80 |
|  |  | Percent change from baseline at Week 12 (n=14) | -6.87 ± 144.52 | -100.00 | -71.18 | 6.49 |
| Abbreviations: IL, interleukin; MMP, matrix metalloproteinase; OPN, osteopontin; SD, standard deviation; TNC, tenascin; TNF, tumor necrosis factor; VCAM, vascular cell adhesion molecule. | | | | | | |

Supplemental Fig. 1 ACR50-CRP response over time.


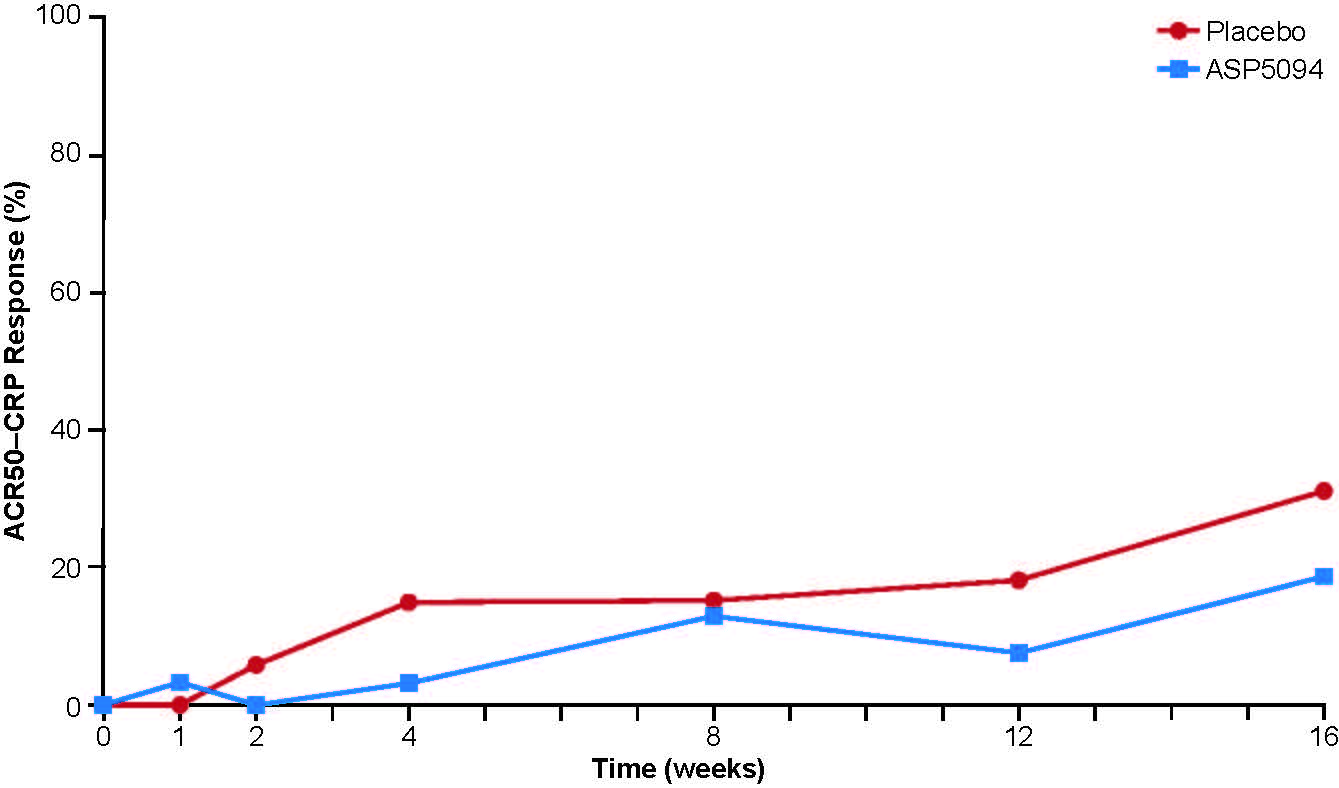


Abbreviation: ACR50-CRP, American College of Rheumatology 50% improvement assessed using the C-reactive protein level.
